# Cytomorphological Studies on Stem of *Luffa echinata* Roxb

**DOI:** 10.4103/0975-1483.66800

**Published:** 2010

**Authors:** S Jayalakshmi, A Patra, AK Wahi

**Affiliations:** *Department of Pharmacognosy, College of Pharmacy, IFTM, Moradabad - 244 001, UP, India*

**Keywords:** Delignification, *Luffa echinata*, pericyclic fiber, vascular bundle

## Abstract

*Luffa echinata* Roxb., commonly known as Bindal in Hindi is used for its hypoglycemic activity in the indigenous system of medicine. No pharmacognostical study on stem is reported in the literature till date; therefore, it was decided to study macroscopical and cytomorphological characters in detail to bring out salient diagnostic features. The stem pieces available in the market are 1.5–17 cm long and 5–8 mm in diameter, showing yellowish-brown to brownish-black surface with longitudinal furrows, fracture is fibrous, and taste is bitter. Mature stem shows single-layered epidermis, seven layers of collenchyma below five ridges but one to two layers of parenchyma in rest of the region beneath the epidermis, continuous wide wavy layer of pericycle composed of three to eight layers of fiber. There are five conjoint bi-collateral open vascular bundles one below each ridge and additional four medullary vascular bundles in the pith each facing furrows.

## INTRODUCTION

*Luffa echinata* Roxb. (Cucurbitaceae) is a climber, commonly known in Hindi as Bindal[1] and is found in Uttar Pradesh, Bihar, Bengal, Sind, Bundelkhand, Dehradun, and in tropical Africa.[2,3] The plant is reported to be emetic, anthelmintic, blood purifier, purgative, antiseptic, antitubercular and useful for anal disease, inflammation, bronchitis, fever, anemia, jaundice, hiccough, and phthisis.[4–7] No pharmacognostical study on stem is on record till date; therefore, the detailed macroscopical and cytomorphological study of stem of *L. echinata* was carried out to bring out the salient diagnostic features, which would enable one to identify the drug available in the market.

## MATERIALS AND METHODS

The samples of *L. echinata* were collected from the local market of Varanasi and authenticated by Dr. V. K. Joshi, Department of Dravyagun, Banaras Hindu University, Varanasi. Free hand sections were taken, stained, mounted as per procedure described by Johansen[8] and other standard methods.[9–11] For studies of isolated tissues and cells, small pieces of stems were macerated in Schulze’s fluid, washed with water, and mounted.[12] Representative diagrams were sketched with the help of camera lucida.

## RESULTS

### Macroscopical characters

**Figure 1-8:**
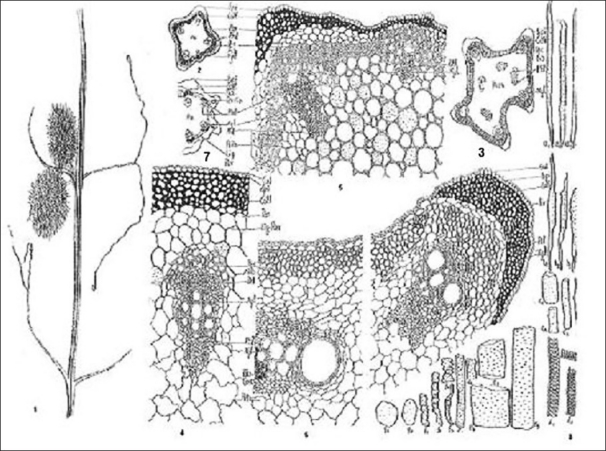
(1) *Luffa echinata* Roxb. (2) T.S. of young stem. (diagrammatic) × 28 (3) T.S. of slightly mature stem. (diagrammatic) × 28 (4) Part of T.S. of Figure 1b showing cellular details × 206 (5) Part of T.S. of Figure 1c showing cellular details × 206 (6) Part of T.S. of Figure 1g showing cellular details × 206 (7) T.S. of mature stem (diagrammatic) × 28 (8) Isolated elements of stem (a_1_-a_4_: Pericyclic fibres, b_1_-b_4_: xylem fibres, c_1_- e_5_: xylem vessels, f_1_- f_4_: Tracheids, g_1_- g_2_: xylem parenchyma)

Stem pieces are slender, yellowish-brown to blackish-brown in color, longitudinally furrowed, glabrous, measuring 1.5–17 cm in length and 5–8 mm diameter. Stems are odorless, bitter in taste, and fracture is short [Figure 1].

### Microscopical characters

The transverse section of young stem shows a quadrangular outline [Figure 2] whereas the mature stem shows five prominent ridges and furrows [Figure 3]. The young stem [Figures 2 and 4] shows single-layered epidermis covered with a thin cuticle and is followed by three to four layers of collenchymatous cells facing ridges and two layers facing furrows showing more prominent angular thickening in cells below ridges. Beneath the collenchyma is one to three cells wide layer of parenchymatous cells. The endodermis is single layered and is followed by sclerenchymatous pericycle two to seven layers wide. Facing each ridge below the pericycle, a large conjoint bi-collateral open vascular bundles is present besides one small developing conjoint collateral vascular bundle facing developing ridge showing few xylem and a few sieve elements. The pith is large consisting of cells, which are lignified showing mostly simple pits except a few which show reticulate thickening.

As the growth proceeds [Figures 5 and 6], the collenchymatous layer becomes discontinuous and the cortex is below the ridges, which is represented by five to eight layers of collenchyma followed by three to four layers of parenchyma. In contrast, in the remaining portion it is represented by one to three layers of collenchyma followed by five to seven layers of parenchyma or only five to seven layers of parenchyma. The pericycle, which appears uniform continuous layer of sclerenchyma, is represented by a layer of highly thickened sclerenchymatous fiber capping the phloem alternating with delignified cells appearing as slightly lignified parenchymatous cells. The pith cells which appear lignified in young stem show delignification and are represented by thin-walled parenchymatous cells varying in shape and size.

The mature stem [Figures 3 and 7] shows a single-layered epidermis covered with a moderately thick cuticle. The cortex is represented by two to eight layers of collenchyma followed by one to three layers of parenchymatous cells in the region facing the ridges and either one to two layers of collenchyma followed by one to two layers of parenchyma or only one to two layers of parenchyma in the remaining portion. Pericyclic cells show less lignification as compared to young or slightly matured stem. The pericyclic fibers are [Figure 8: a_1_, a_3_, a_4_] long, narrow with pointed to blunt ends showing wide lumen and simple pits on their walls. Some of the fibers show beak like ends [Figure 8: a_2_]. Similar to normal vascular bundles, four medullary vascular bundles develop in the pith region each facing the furrows. Some of the parenchymatous cells of phloem show tanniferous content. The xylem fibers are distinctly smaller in size as compared to pericyclic fiber [Figure 8: b_1_–b_4_]. Some of these fibers showed notch projection on one or both the ends. The vessels are cylindrical drum shaped with border on their walls [Figure 8: c_1_ –c_4_, e_1_–e _5_]; however, some vessels [Figure 8: d_1_–d_4_] show spiral thickening as well as tracheids [Figure 8: f_1_–f_4_] vary in shape and show simple pits on their walls and are septed. The xylem parenchyma is also thickened showing simple pits on their walls [Figure 8: g_1_, g_2_].

Measurement of different cells and tissues of the stem of *L. echinata* Roxb. are given below in microns:

Epidermis: 28 − 53.6 × 84 × 21 − 49 − 84

Collenchyma: 14 × 57.16 × 112 × 28 − 58.3 − 98

Parenchyma: 35 − 57.75 − 98 × 84 − 117.8 − 154

Pericyclic fiber: 200 × 536.4 − 937.5 × 6.25 − 14.77 − 25

Xylem fiber: 162.5 − 340.1 − 593.7 × 12.5 − 21.8 − 25

Xylem vessels: 87.5 − 255.76 − 562 × 12.5 − 37.5 − 87.5

Xylem parenchyma: 75 × 87.5 − 112.5 × 37.5 − 57.5 − 87.5

Pith cells: 112 − 204.16 − 336 × 70 − 164.5 − 364

## DISCUSSION

The stem can be identified by its yellowish-brown to blackish-brown color and longitudinal furrows besides young stem microscopically can be identified by the presence of quadrangular outline, parenchyma followed by a continuous layer of collenchyma, thick and lignified pericyclic fiber, four conjoint bi-collateral vascular bundles and pith with thick-walled and pitted cells. Similarly mature stems can be characterized by its five distinct ridges and furrows, less-thickened pericyclic sclerenchyma, five conjoint bi-collateral vascular bundles facing the ridges, and four medullary bundles facing the furrows and thin-walled and delignified pith cells.
